# Novel MYO15A variants are associated with hearing loss in the two Iranian pedigrees

**DOI:** 10.1186/s12881-020-01168-x

**Published:** 2020-11-18

**Authors:** Somayeh Khatami, Masomeh Askari, Fatemeh Bahreini, Morteza Hashemzadeh-Chaleshtori, Saeed Hematian, Samira Asgharzade

**Affiliations:** 1 Department of Genetics and Molecular Medicine, School of Medicine, Zanjan University of Medical Sciences, Zanjan, Iran; 2 Department of Genetics at Reproductive Biomedicine Research Center, Royan Institute for Reproductive Biomedicine, ACECR, Tehran, Iran; 3 Department of Molecular Medicine and Genetics, Faculty of Medicine, Hamadan University of Medical, Hamadan, Iran; 4 Cellular and Molecular Research Center, Basic Health Sciences Institute, Shahrekord University of Medical Sciences, Shahrekord, Iran

**Keywords:** *MYO15A*, Whole exome sequencing, First approach, Consanguineous

## Abstract

**Background:**

Clinical genetic diagnosis of non-syndromic hearing loss (NSHL) is quite challenging. With regard to its high heterogeneity as well as large size of some genes, it is also really difficult to detect causative mutations using traditional approaches. One of the recent technologies called whole-exome sequencing (WES) has been thus developed in this domain to remove the limitations of conventional methods.

**Methods:**

This study was a report on a research study of two unrelated pedigrees with multiple affected cases of hearing loss (HL). Accordingly, clinical evaluations and genetic analysis were performed in both families.

**Results:**

The results of WES data analysis to uncover autosomal recessive non-syndromic hearing loss (ARNSHL) disease-causing variants was reported in the present study. Initial analysis identified two novel variants of *MYO15A* i.e. *c.T6442A:p.W2148R* and c.10504dupT:p.C3502Lfs*15 correspondingly which were later confirmed by Sanger validations and segregation analyses. According to online prediction tools, both identified variants seemed to have damaging effects.

**Conclusion:**

In this study, whole exome sequencing were used as a first approach strategy to identify the two novel variants in *MYO15A* in two Iranian families with ARNSHL.

## Background

Hearing impairment is considered as an etiologically heterogeneous sensory deficiency with incidence 1 in 1000 newborns around the world [1]. In this regard, genetic hearing loss (HL) has been divided into syndromic and non-syndromic types. Considering the high rate of consanguineous marriages in the Middle East, autosomal recessive non-syndromic hearing loss (ARNSHL) is reportedly more prevalent western countries [2]. However; due to the wide variety of pathogenic genes associated with non-syndromic hearing loss (NSHL), including both nuclear and mitochondrial ones, the disease includes diverse patterns of inheritance comprised of autosomal dominant, autosomal recessive, mitochondrial, and X-linked recessive. Disease heterogeneity has been admittedly recognized as the most important challenge in genetic diagnosis of NSHL. Diagnostic approaches which have been relied on conventional methods based on genetic testing of the most common genes, often fail to determine the exact genetic cause of the disease in many countries including Iran [3]. In heterogeneous populations like Iran, the distribution of mutations in the gap junction beta-2 protein, also known as connexin 26, (i.e. *GJB2*) gene as a major cause of ARNSHL can be extremely diverse depending on patients’ ethnicities. The prevalence rate of *GJB2*-related hearing loss has been reported by 38.3% in northern Iran, but the percentage of such variations has been found very rarely in southern regions [3]. Various frequencies of causative mutations, compound heterozygotes, as well as nuclear modifier genes can also render the molecular diagnosis of ARNSHL as a challenge [3, 4]. Moreover, mutation screening in large genes such as *MYO15A* (66 exons) can be impeded following the use of traditional approaches based on Sanger sequencing [5, 6]. Today, molecular genetic testing on the basis of multi-gene screening such as whole-exome sequencing (WES) are being used instead of traditional diagnostic procedures [7].

For first time, *MYO15A* (DFNB3 locus) mutation was reported from Indonesia population. Until now, there have been many reports of mutations of *MYO15A* causing ARNSHL in different countries of Asia such as Pakistan [8], Turkey [9] and Iran [6]. In the present study, two novel *MYO15A* variants identified by WES from two Iranian families with ARNSHL is reported.

## Methods

### Subjects and clinical evaluations

The study was approved by the Ethics Committee of Shahrekord University of Medical Sciences (IR.SKUMS.REC.1397.008), Iran. Two Iranian families from Hamedan Province with hearing impairments, without any other additional symptoms were thus studied. Informed written consent was taken from both families. The proband from each family was further subjected to clinical evaluations of the inner ear accompanied by pure-tone audiometry (PTA).

### Molecular analysis

WES was used to detect the deafness associated variants in the DNA sample in probands. Genomic deoxyribonucleic acid (DNA) was extracted from whole peripheral blood of each study subject utilizing DNA Extraction Kit DNP (Sinacolon, Iran) according to the manufacturer’s instructions. Purity and concentration of DNA samples were further measured via Thermo Scientific NanoDrop 2000c Spectrophotometer.

**Fig. 1:**
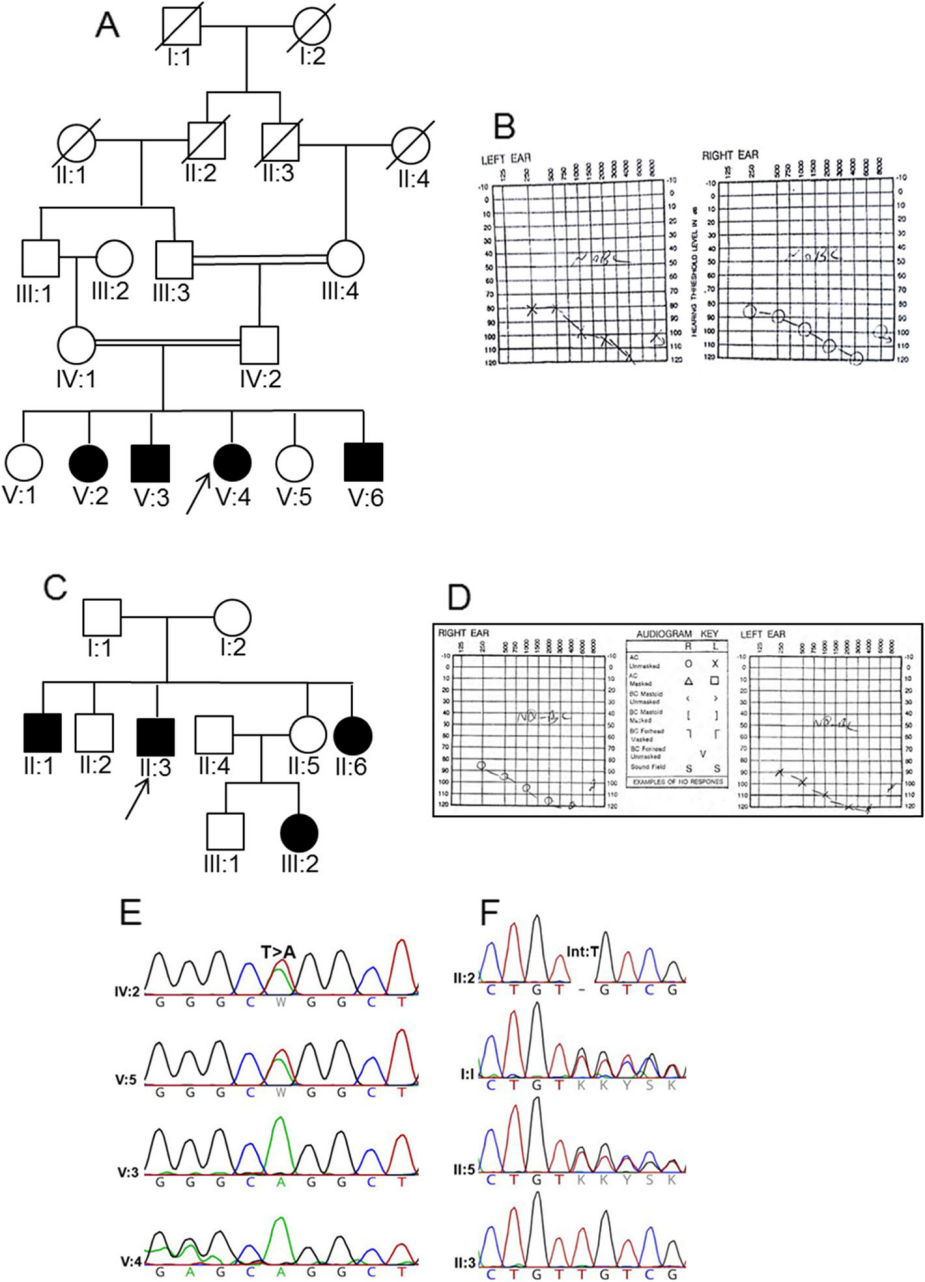
Pedigrees and the proband audiograms, as well as sequencing chromatograms. **a** Pedigree of family 1 having autosomal recessive form of NSHL disease is drawn. The proband (V: 4), for whom whole exome Sequencing has been carried out is indicated by arrow. **b** Audiogram for pure tone audiometry (PTA) of the proband (V: 4) with profound hearing loss in both ears. **c** Three-generation pedigree of family 2 having autosomal recessive form of NSHL disease is drawn. The proband (II: 3), for whom whole exome Sequencing has been carried out is indicated by arrow . **d** Audiogram for pure tone audiometry (PTA) of left and right ears of the affected proband (II: 3) showed profound hearing loss. Hearing impaired individuals are illustrated by black-filled symbols. **e** Partial sequence chromatograms of *MYO15A* gene from two unaffected (IV.2 and V.5) and affected individuals (V: 3 and V: 4) in family 1 illustrate T to A transition at position 6442. **f** Partial sequence chromatograms of *MYO15A* gene containing (c.10504dupT:p.C3502Lfs*15) variant in proband (II: 3), his father (I: 1) and his siblings (II: 2 and II:5) in family 2 illustrates insertion of T as indicated

DNA samples from each pedigree’s proband (Fig. 1a, V-4 in family 1; Fig. 1c, II-3 in family 2) were then subjected to WES at Macrogen Online Sequencing Order System (Seoul, South Korea) on Genome Analyzer/HiSeq 2000 (Illumina, San Diego, CA, USA, 151-bp paired-end reads). It should be noted that the library had been prepared through SureSelect XT Library Prep Kit (Agilent Technologies, CA, USA). Data analysis was correspondingly performed using an in-house developed pipeline, adopted from Genome Analysis Tool Kit v3.6 and ANNOVAR software [10]. Homozygous missense, start codon change, splice site, nonsense, stop loss, and indel variants with minor allele frequency < 1% were further filtered in dbSNP (version 138), 1000 Genomes Project, Exome Aggregation Consortium (ExAC), and NHLBI GO Exome Sequencing Project (ESP). Based on autosomal recessive inheritance, the homozygosity region of samples was determined using homozygosity mapping algorithms.

In order to prioritize the candidate functional variants, several online prediction software including MutationTaster2, FATHMM, PANTHER, SIFT, PROVEAN, MetaLR, PolyPhen-2, CADD, and ConSurf were also used to evaluate the pathogenic effects of the variants with a frequency less than 0.01. After observing the autosomal recessive inheritance pattern in both pedigrees, the homozygous variants were then prioritized. Next, the variants were investigated in the Human Gene Mutation Database, hereditaryhearingloss home page and the related literature to survey their association with a phenotype and novelty of the variants.

Besides, candidate variant segregation from exome data was evaluated through polymerase chain reaction (PCR)-based Sanger sequencing. Therefore, the following primers were synthesized: *5ʹ-GAACTACATCGTGCAGAAGG-3ʹ* and *5ʹ-CCTATCCAGTCCCACTCACT-3ʹ* for human *MYO15A c.T6442A* variant and 5ʹ-*CCACCATTCGGCCTTCCA-3ʹ* and *5ʹ-CTGCCTCCTCTTAGTGTCCTC-3ʹ* for human *MYO15A c.10504dupT* variant.

## Results

### Clinical and molecular findings

#### Family1

The first family pedigree is displayed in Fig. 1a. Accordingly, four members of the pedigree including two affected and two unaffected individuals who consented to be included in this study are indicated. In this family, the proband (V: 4) was a 21-year-old woman with congenital HL. No additional abnormal phenotypic features including visual impairments or any limb and facial malformations were observed in the proband. The parents were consanguineous and both showed normal hearing. According to the audiogram, the proband is suffering from congenital profound deafness (Fig. 1b).

#### Family2

A three-generation pedigree, depicted in Fig. 1c, was presented as the second family with ten members, six males and fourteen females, suffering from ARNSHL. The proband was a 25-year-old male individual, born as the second child of non-sanguineous healthy parents, who had been diagnosed for congenital HL when he was 1 year old. At the age of 5, he had gone through bilateral cochlear implant surgery based on physical examinations and audiometry testing. Audiogram analysis also confirmed HL to be profound in the proband (II: 3) (Fig. 1d).

**Fig. 2:**
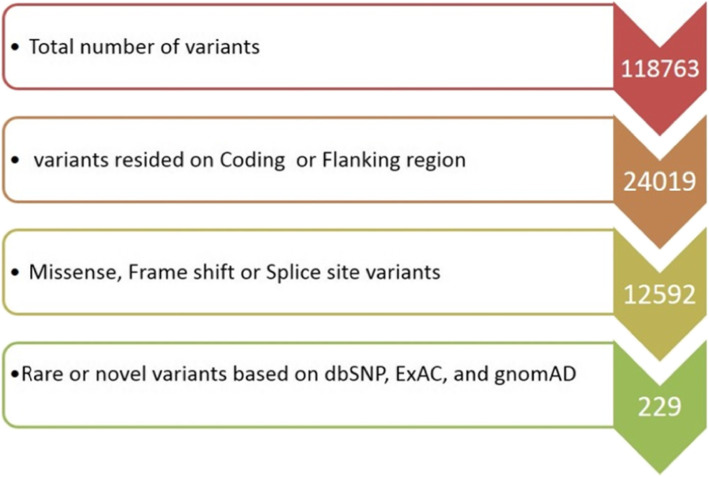
Schematic flow chart of the filtering of causative variants in this study

**Fig. 3:**
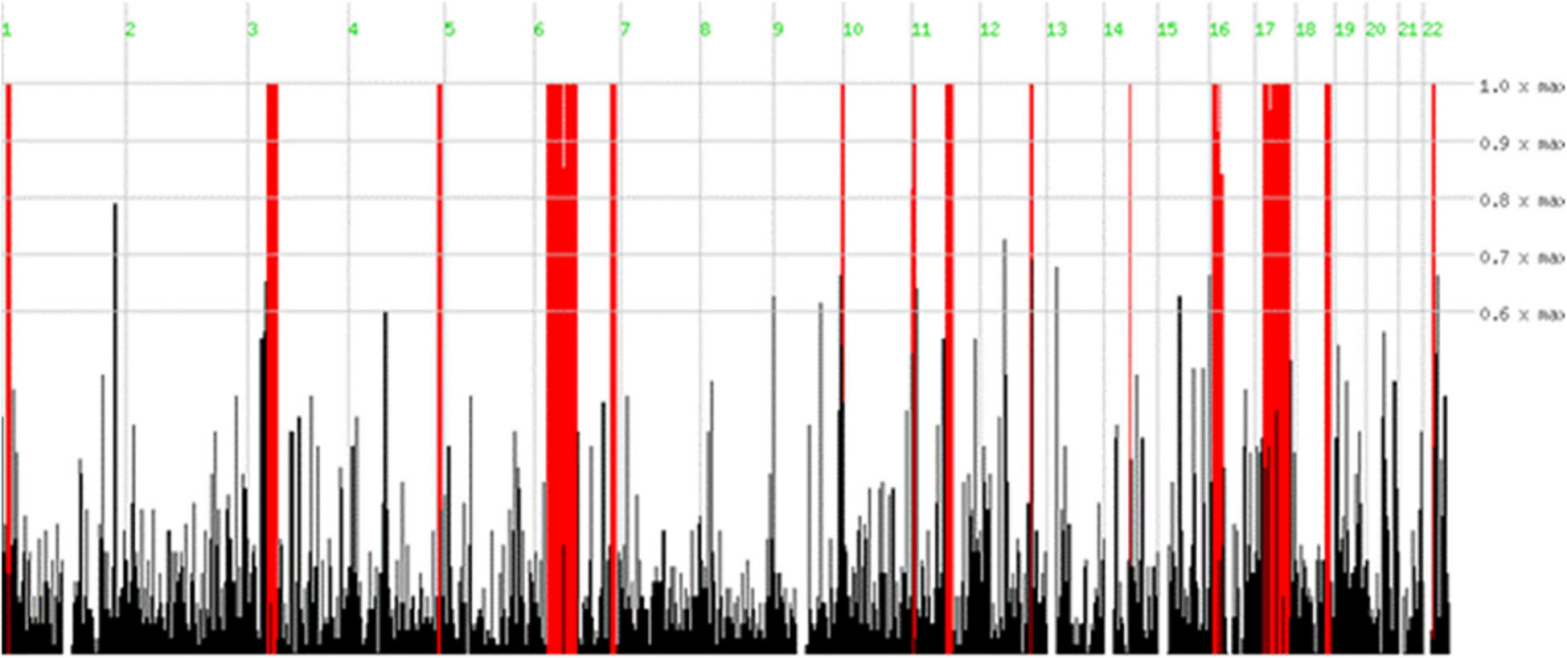
Homozygosity region in the proband (V: 4) of pedigreeI. Coordinate homozygosity region on chromosome 17 in proband [17927849–18,239,689]

Considering the all limitations in genetic diagnosis of ARNSHL, it was decided to perform WES as a first approach on proband’s genome DNA. The total number of bases, reads, GC (%), and Q30 (%) are calculated for the subjected sample. 70,760,838 reads were produced, and total read bases were 10.7G bp. The GC content (%) and Q30 were 51.87 and 95.69% respectively. Following the filtering step depicted in Fig. 2, two novel homozygous variants in *MYO15A* i.e. *c.T6442A:p.W2148R* and c.10504dupT:p.C3502Lfs*15 were prioritized in family I and II, respectively. Given that the proband of family I was the offspring of consanguineous parents, WES dataset revealed that *MYO15A* (*c.T6442A*) variants had resided in the large homozygous regions on chromosome 17 (Fig. 3). All in silico programs also predicted damaging effects of *p.W2148R* variant. Mutation taster further suggested that *c.10504dupT* variant had deleterious effects. Moreover, the first conservative amino acid alternation (*p.C3502L*) predicted to be pathogenic using online softwares (Table 1). Analysis of genotype-phenotype correlations revealed that patients with c.T6442A:p.W2148R and c.10504dupT:p.C3502Lfs*15 tended to have profound hearing loss.

**Table 1:** In Silico and Bioinformatics Analysis of the Variants

Variant	c.T6442A:p.W2148R	c.10504dupT:p.C3502Lfs*15
Locus	DFNB3	DFNB3
dbSNP rsID	Novel	Novel
ConSurf score	8	8
MutationTaster2	Disease causing	Disease causing
SIFT	Damaging	deleterious
Polyphen2	Probably damaging	Probably damaging
FATHMM	Damaging	Damaging
PROVEAN	Deleterious	Deleterious
MetaLR	Damaging	Not down
CADD_phred	24.3	Not down
PANTHER	Probably damaging	Probably damaging
Segregates in the family	Yes	Yes

*Creating a new reading frame ending at a stop codon at position 15

Additionally, the results of Sanger sequencing confirmed the presence of *p.W2148R* variant in *MYO15A* gene in the proband and other affected members who were studied, but the unaffected sister (V.5) and her father were found heterozygous for the variant (Fig. 1e).

The findings of Sanger sequencing also revealed co-segregation of *c.10504dupT* variant in the second family (Fig. 1f). The affected proband was thus homozygous, whereas his unaffected sister (II:5) and his father (I:1) were heterozygous for this locus and his unaffected brother (II:2) lacked the variant.

It should be noted that these two variants were absent in 50 ethnically-matched control cases.

## Discussion

Based on WES data as well as segregation and genotype-phenotype correlational studies, mutations in MYO15A gene were identified as a main contributor of NSHL in the first and second surveyed families.

*MYO15A*, as a new branch of the myosin protein-coding gene superfamily, has a role in stereocilia formation [11]. This gene is considered as the third leading cause of ARNSHL in many populations [12, 13], including Iran, with a prevalence rate ranged from 4.8–9.6% [14–16] . Moreover, mutations in this gene have been associated with severe-to-profound HL. Screening of 66 coding exons through Sanger sequencing is expensive and more time-consuming. On the contrary, high-throughput techniques can save time and money [17].

MYO15A is a different form of myosins protein with long N-terminal extensions following by the conserved motor domain, IQ motifs (calmodulin/ myosin light chain binding), MyTh4 domains (Myosin-Tail like Homology region 4), FERM motifs (4.1 protein, Ezrin, Radixin, and Moesin), SH3 domain (Src Homology 3), and the PDZ ligand domain (Post synaptic density protein (PSD95), Dlg1 (Drosophila disc large tumor suppressor), and zo-1 (Zonula occludens-1 protein) [18]. The identified *c.T6442A: p.W2148R* variant in our study is located in the first (MyTH4) domain and is also conserved among different species (data not shown). The association of (MyTH4) domain mutations with hearing loss was firstly reported in 1998 [19]. Due to documents, variants in this domain have related severe to profound hearing loss, which is consistent with our pedigree phenotype [5, 15, 16, 20–22]. To the best of our knowledge, p.P2073S, p.R2124Q [22] p.P2073L, p.V2114G [16], R2146Q [23], p.R2146W [24] and c.6273 + 1G > A [25] variants have been detected in Iranian population until now [15, 16, 22]. The p.R2124Q and p.P2073S were the first reported mutations in the MyTH4 domain of MYO15A protein which was located in conserved fourth helix of MyTH4. Variations in the MyTH4 domains interfere in forming of transmembrane actin microfilament assembly complex at the stereocilia tips [22].

Mehregan et al., reported p.Arg2146Gln in the fourth helix of the first MyTH4 core, which results in severe to profound hearing loss. Structural analysis of this variation has revealed that this substitution alters binding properties at the domain surface [24].

The substitution of a highly conserved amino acid (Table 1), hydrophobic non-polar tryptophan, with arginine can lead to a loss of hydrophobic pocket. The counterparts of p.W2148 in the more studied family members, i.e. MYO7A is W1192. Sans CEN2 (a scaffold protein) interacted with MyTH4 domain by extensive hydrogen bonding, hydrophobic contacts, and charge-charge interactions. The hydrophobic pocket in MYO7A comprised the conserved A1189, W1192, I1193, P1220, and Y1223 residues. Substitution of Ala1189 by Glu, leads to a.

~ 10-fold decrease of the binding affinity between MyTH4 domain and CEN. The complex of myosins and SANS linked cadherins to the actin cytoskeleton [26]. Woo et al., explained that p.R2146Q in myosin 15A and R1190 of MYO7A had similar structure in MyTH4- FERM domains and interfere to binding CEN2 to this domain [5].

In addition, Myosin interacts with other scaffolding proteins (whirlin and Eps8 (Epidermal Growth Factor Receptor Pathway Substrate 8)) and can transport them to the tip of stereocilia to form a stereocilia tip complex, which can facilitate maturation of stereocilia. These scaffold proteins are essential for normal hearing in humans [27, 28]. MyTH4 domain contains the actin-binding sites. The overall surface of the microtubule (MT) is negatively charged. The positively charged motifs with surface-exposed hydrophobic side chains found in the myosin MyTH4 domain can serve as an MT binding site [29].

We speculate the, *c.T6442A:p.W2148R* variant interferes with the formation of the Myosin 15A-whirlin-Eps8-CEN2 complexs and microtubule-binding.

In the second studied family, WES could successfully detect a novel homozygous insertion variant i.e. c.10504dupT:p.C3502Lfs*15 in *MYO15A* gene, co-segregated with the disease within the pedigree. This variant between the second FERM and PDZ domains could also lead to a reading frame shift at position 10,504 and a stop codon (p.C3502Lfs*15) with truncation and translation of mRNA resulting in lack of its conserved amino acids at C-terminal (data not shown). Lezirovitz et al. reported a frameshift mutation c.10573delA to cause profound.

hearing loss. This mutation in the PDZ binding ligand of MYO15A altered interaction of this protein with whirlin. Thus, stereocilia elongation did not occurred [30].

Zhang et al. identified p.Leu3501Glu variant was associated with profound hearing loss [17].

Today, the topic of oligogenic inheritance traits has made a big wave in diagnostic medicine; since, in many monogenic diseases, it represents that there is not just one gene affected phenotype which causes new challenges in diagnosing these diseases and it can be more complicated for diseases with heterogenic pathology in which many genes are involved [31].

## Conclusion

In summary, we identified two novel variants (p.W2148R and p.C3502Lfs*15), in the MYO15A gene in two Iranian families using whole-exome sequencing. Accordingly, we showed that these likely pathogenic variants were segregated with the profound hearing loss in both families. However, further functional analysis is required to confirm the results of the present study.

## Data Availability

The raw datasets generated and/or analyzed during the current study are not publicly available because it is possible that individual privacy could be compromised, but are available from the corresponding author on reasonable request.

## Abbreviations

AR: Autosomal recessive
ExAC: Exome Aggregation Consortium
ESP: Exome Sequencing Project
ESP8: Epidermal Growth Factor Receptor Pathway Substrate 8
FERM: 4.1 protein, Ezrin, Radixin, and Moesin
MyTh4: Myosin-Tail like Homology region 4
NSHL: Non-syndromic hearing loss
PTA: Pure-tone audiometry
SH3: Src Homology 3
WES: Whole exome sequencing
zo-1: Zonula occludens-1 protein
